# The Effects of Augmented Eccentric Loading upon Kinematics and Muscle Activation in Bench Press Performance

**DOI:** 10.3390/jfmk5010008

**Published:** 2020-01-26

**Authors:** Roland van den Tillaar, Kedric Kwan

**Affiliations:** 1 Department of Sport Sciences and Physical Education, Nord University, 7600 Levanger, Norway; 2 Sports Performance Research Institute New Zealand (SPRINZ), Auckland University of Technology, Auckland 0632, New Zealand; kedric.kwan@aut.ac.nz

**Keywords:** potentiation, peak velocity, EMG, augmented eccentric, explosive bench press

## Abstract

The aim of this study was to investigate the effects of an augmented eccentric load upon the kinematics and muscle activation of bench press, and to investigate possible mechanisms behind augmented eccentric loading during the lift. Sixteen resistance-trained males (age 28.5 ± 7.7 years, height 1.78 ± 0.08 m, body mass 80.7 ± 14.3 kg) performed three repetitions at 95/85% of 1RM (augmented eccentric loading), and 85/85% of 1RM (control) in bench press, while barbell kinematics and muscle activation of eight muscles were measured. The main findings were that no kinematic differences between the augmented and control condition were found, only an effect of repetition. Furthermore, augmented loading caused a higher activation of the biceps brachii during the pre-sticking and sticking region, while a lower activation in the sternal part of pectoralis major during the eccentric phase was observed. Based on the present findings, it can be concluded that augmented eccentric loading with 95% of 1RM in bench press did not have any acute positive effect upon the concentric phase of the lift (85% of 1RM) and that the proposed underlying mechanisms like potentiation, increased neural stimulation and preload, and recovery of stored elastic energy does not seem to occur with these loads.

## 1. Introduction

In general, a muscle undergoes two phases during contraction; the eccentric (muscle lengthening) phase followed by the concentric (muscle shortening) phase. Multiple studies [1,2,3,4] have shown that performing an eccentric action prior to a concentric action positively influences the concentric execution, which is known as the stretch shortening cycle (SSC). It has been commonly found that a vertical jump following a countermovement will result in a higher vertical distance compared to one without [5]. In addition, by increasing the loading during the eccentric phase it can further increase vertical jump height, such as in the depth jump [6]. Numerous studies have also compared the efficacy of a loaded eccentric phase using an additional 20–30% of body mass on a drop jump, which also led to an increase in subsequent jump height in (highly) trained athletes [7,8].

While the mechanism of an improved concentric action is still not fully understood, several have been proposed [9]. Higher loading during the eccentric phase may increase force production due to an enhanced synaptic excitation in the spinal cord. This leads to greater post synaptic potential, increasing the force production capacity of the involved muscle groups, which is known as potentiation [10]. Besides that, a greater eccentric load may lead to changes in muscle recruitment. Nardone; Romano; Schieppati [11] demonstrated that during eccentric contractions, selective recruitment of high threshold motor units was possible and that this lead to greater concentric force production by the contribution of larger motor unit pools. In fact, Walker; Blazevich; Haff; Tufano; Newton; Hakkinen [12] reported that an increase in voluntary muscle activation in the vastus lateralis following an augmented eccentric load led to greater isometric strength compared to traditional loading after a 10-week protocol in strength-trained men.

In bench press with resistance-trained men, utilization of the SSC was demonstrated by Sakamoto; Sinclair [13], who reported that more repetitions in the bench press exercise were completed at a given intensity press when a higher execution speed was utilised. In addition, Doan; Newton; Marsit; Triplett-McBride; Koziris; Fry; Kraemer [14] found that concentric performance in the bench press improved by ≈2–7 kg following an augmented eccentric loading compared to traditional loading. In addition, Sheppard; Young [15] found significant increases in barbell distance on the bench throw when greater eccentric loads were used. However, Ojasto; Hakkinen [16] reported a detrimental effect on maximal strength expression after an augmented eccentric load protocol in bench press using 100, 105, 110 and 120% of the concentric 1RM. The discrepancy could be postulated to different loads used during the eccentric bout of the lift. Furthermore, to our knowledge, no study had yet investigated the effects of an augmented eccentric loading on subsequent concentric performance in the bench press and possible mechanisms (potentiation, higher muscle activation, preload, and recovery of stored elastic energy) could explain the enhancement of the concentric phase, as previous studies investigated only the outcome and not the underlying possible explaining mechanisms behind the outcomes. Therefore, the aim of this study was to investigate the effects of an augmented eccentric load upon the kinematics and muscle activation that occur during bench press, and to investigate possible mechanisms behind the eventual enhancement during the concentric phase. It was hypothesised that augmented eccentric loading was more effective on the concentric phase (higher barbell velocities and later occurrence of the sticking region) than performing traditional bench press (same load, eccentric–concentric) due to the enhanced effects of potentiation and muscle activation [1,4].

## 2. Materials and Methods

### 2.1. Participants

Sixteen resistance-trained males (age 28.5 ± 7.7 years, height 1.78 ± 0.08 m, body mass 80.7 ± 14.3 kg) recruited from a local training centre and power lifting club volunteered to participate in the study. Participants were excluded from the study if they had muscular pain, illness or injuries that could reduce their capacity to perform bench press with maximal effort. The participants were recruited on the basis that they had at least a year of experience with general resistance training, were familiarized with the bench press exercise, and were able to perform a one-repetition maximum (1RM) equal to their body mass (kg). The participants were instructed to refrain from any additional resistance training targeting the upper body during the 48 h before testing. Each participant was informed of the testing procedures and possible risks, and written consent was obtained prior to the study. The study complied with the current ethical regulations for research and was approved by the Norwegian Centre for Research Data (project 42440, 27 Feb 2015), conformed to the latest revision of the Declaration of Helsinki.

### 2.2. Procedure

To calculate the loads for the experiment, a separate session was conducted approximately one week before the main test, in which 1RM of each participant was established. In the same session, all participants were familiarized with the laboratory equipment and the augmented eccentric loading task using weight releasers. One week after their 1RM testing, each subject performed on the experimental day both the augmented eccentric loading protocol and the control. Prior to the familiarization, each participant performed a standardized warm up of ten repetitions at 50% of estimated 1RM, five repetitions at 70% of estimated 1RM, and three repetitions at 80% of estimated 1RM, followed by several attempts to determine their actual 1RM. If the participant was successful or unsuccessful in lifting the load, they were given a 3–4 min rest period before another maximal attempt was performed with 2.5–5% higher/lower load than the previous attempt until they reached their 1RM (this was often found in two to three attempts). On the experimental day, the same standard warm-up with the percentage of actual 1RM was used, followed by the control or augmented eccentric loading protocol.

The test protocols were conducted on the same day to ensure that all sensors were on the same place and with a one-hour rest between them to prevent fatigue. It was a counterbalanced crossover design, which means that half of the subjects started with the augmented eccentric loading protocol, while the other half started with the control condition. The augmented eccentric loading protocol consisted of three repetitions at 95/85% of 1RM (eccentric/concentric), and a control conditioning protocol of three repetitions at 85/85% of 1RM (equal load) in the bench press exercise. A pilot study with four subjects was conducted to examine how heavy the load could be for three repetitions to be lifted using 10% extra weight in the eccentric phase. In the pilot, the subjects were able to lift three repetitions using 95% 1RM in the eccentric phase and 85% 1RM in the concentric phase. Therefore, these loads were chosen for the present study.

To create a higher eccentric resistance than concentric resistance in the bench press exercise, specially designed weight releasers (Getstrength, Waiuku, New Zealand) were used. Weight releasers have the potential to maximize the resistance in the eccentric phase and reduce it in the concentric phase of the movement [16]. The weight releaser was detached from the barbell at the end of the eccentric phase and the concentric phase was performed with a lower resistance. After detachment, the weight releasers remain on the floor after one repetition. Therefore, two assistants return the weight releasers on the barbell for consecutive repetitions. To assure a similar lifting procedure between the two conditions, a pause of approximately 2–3 s between each of the three lifts was conducted.

### 2.3. Measurements

Before testing, the skin on which the electrodes were placed was first shaved, washed with ethanol, and abraded to reduce impedance. The electrodes (11 mm contact diameter and a 2 cm center-to-center distance) were placed along the presumed direction of the underlying muscle fiber according to the recommendations of SENIAM [17], and as used in other studies [18,19,20]. To strengthen the signal, conductive gel was applied to self-adhesive electrodes (Dri-Stick Silver circular sEMG Electrodes AE-131, NeuroDyne Medical, Cambridge, MA, USA). EMG activity was measured with Musclelab v10.5.67 software (Ergotest Technology AS, Porsgrunn, Norway). The electrodes were placed on the right upper limb and positioned on the belly of the sternal and clavicular part of the pectoralis major, the anterior and medial deltoid, the lateral, medial, long head of the triceps brachii, and the biceps brachii. The raw EMG signals, sampled at 1000 Hz were amplified and filtered using a preamplifier located as close to the pickup point as possible. The EMG signals were converted to root mean square (RMS) EMG signals using a hardware circuit network (frequency response 20–500 kHz, averaging constant 100 ms, total error ± 0.5%). The mean RMS EMG signals of each muscle during the downward and upward phase of the lift in each condition were used for further analysis.

**Figure 1:**
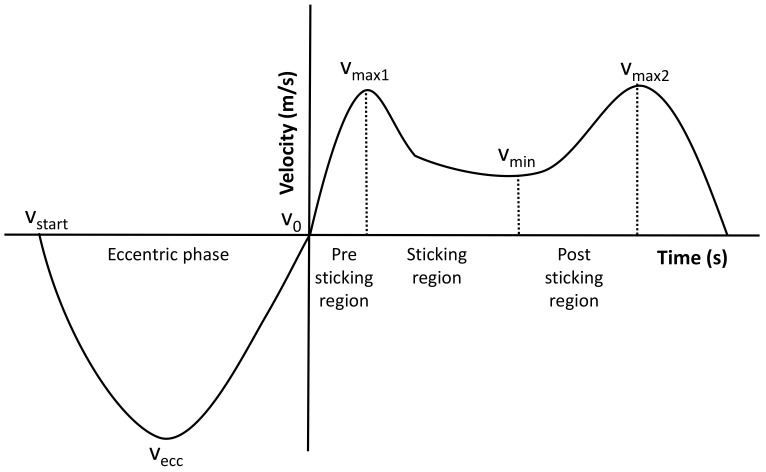
Representative velocity–time curve during (sub) maximal bench press lifts ( > 85% of 1RM) with the different events and regions [21,23].

To locate possible differences in muscle activity during bench press movement between the two conditions, the average root mean square was calculated for each of the three regions in the ascending part of each repetition. The first region was from the eccentric phase of the lift. The second region was from the lowest barbell point where the velocity is zero (v0) to the first maximal barbell velocity (v_max1_): the pre-sticking region. The second region was from the maximal barbell velocity until the first located lowest barbell velocity (v_min_): the sticking region. The last period, the post-sticking region, began at the first located lowest barbell velocity and went to the second maximal barbell peak velocity (v_max2_), which was also called the strength region (Figure 1) [21]. No normalization of the EMG signals was necessary as all measurements per participant were performed in one session and only a within-subject design was used [19,22]. The phase duration for mean RMS EMG analysis was defined using the linear encoder.

A linear encoder (ET-Enc-02, Ergotest Technology AS, Langesund, Norway) connected to the barbell measured barbell distance and velocity over time with a resolution of 0.019 mm and sampling rate of 200 Hz. The vertical distance was measured in relation to the lowest point of the barbell (zero distance). The velocity of the barbell was calculated by using a five point differential filter with Musclelab v10.5.67 software (Ergotest Technology AS, Langesund, Norway). From each lift the peak velocity in eccentric phase (v_ecc_) and the events: v_max1_, v_min_ and v_max2_ of the concentric phase in each condition were used for further analysis, together with the timing of these events. The linear encoder and EMG signals were synchronized through the Musclelab 6000 system.

### 2.4. Statistical Analysis

To assess differences in barbell kinematics (velocity, distance and time) and EMG activity between the two conditions, a repeated 2 (Condition: augmented eccentric loading vs. Control) × 3 (repetition 1–3) model analysis of variance (ANOVA) design was used for the different events (v_start_, v_ecc_, v_max1_, v_min_ and v_max2_) and regions (eccentric, pre-sticking, sticking and post-sticking). Data was checked for normality with the Shapiro–Wilks test. If significant differences were found, a Holm–Bonferroni post-hoc test was performed. In cases where the sphericity assumption was violated, the Greenhouse–Geisser adjustments of the *p*-values were reported. The level of significance was set at *p* ≤ 0.05. Statistical analysis was performed with SPSS version 24.0 (SPSS Inc, Chicago, IL, USA). Effect size was evaluated with η^2^ (Eta squared) where 0.01 < η^2^ < 0.06 constitutes a small effect, 0.06 < η^2^ < 0.14 constitutes a medium effect, and η^2^ > 0.14 constitutes a large effect [24].

## 3. Results

**Figure 2:**
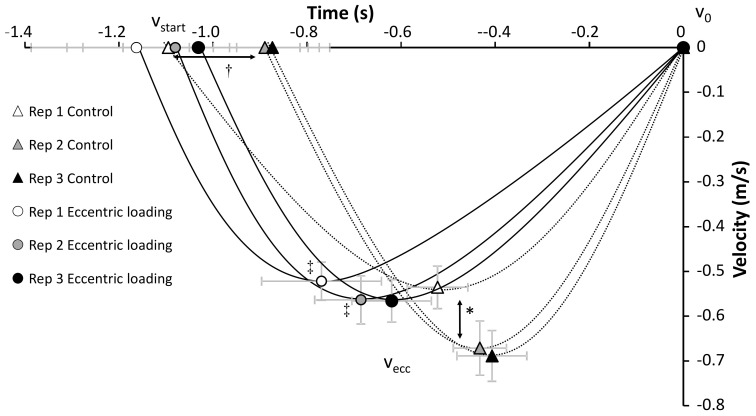
Peak mean velocities (± SEM) at the different events in each repetition for each condition during the eccentric phase. * indicates a significant difference in mean peak velocity for these two repetitions with all other repetitions at *p* < 0.05. † indicates a significant difference in timing for this repetition with the others from this condition at *p* < 0.05. ‡ indicates a significant difference in timing for this repetition with the same repetition from the other condition at *p* < 0.05.

**Figure 3:**
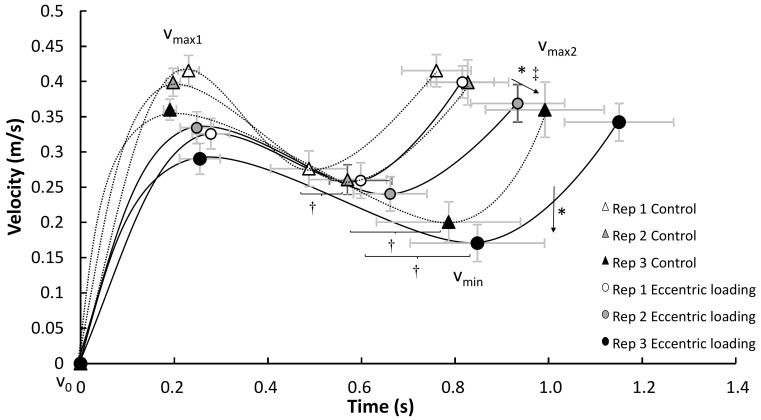
Peak mean velocities (± SEM) at the different events in each repetition for each condition during the concentric phase. * indicates a significant decrease in mean peak velocity (v_min_ and v_max2_) between repetition 3 with all other repetitions in both conditions on a *p* < 0.05. ‡ indicates a significant difference in time of occurrence of peak velocity between repetition 3 with the others in both conditions at *p* < 0.05. † indicates a significant difference in time of occurrence of peak velocity between these two repetitions in this condition at *p* < 0.05.

Only a significant effect of condition was found for the peak eccentric velocity (F = 5.4, *p* = 0.034, η^2^ = 0.27) and time of occurrence of this event (F = 6.9, *p* = 0.019, η^2^ = 0.31) with a lower peak eccentric velocity occurring earlier for the augmented eccentric condition compared with the control condition. Furthermore, an effect was found for repetition in peak eccentric velocity, minimal concentric velocity and second peak velocity (F ≥ 16.6, *p* ≤ 0.001, η^2^ ≥ 0.62) and time of occurrence of the minimal concentric velocity and second peak velocity (F ≥ 7.0, *p* ≤ 0.023, η^2^ ≥ 0.41). In addition, an interaction of condition*repetition was found for the peak eccentric velocity (F = 4.7, *p* = 0.037, η^2^ = 0.24). Post hoc comparisons revealed that the total eccentric phase was significantly longer in repetition 1 compared to the other repetitions in the control condition. The peak eccentric velocity was significantly higher in repetition 2 and 3 in the control condition compared with all others and the time of occurrence of peak eccentric velocity appeared earlier in repetition 1 and 2 in eccentric loading compared with the control condition (Figure 2). In the concentric phase, minimal concentric velocity and second peak velocity were significantly lower in repetition 3 compared with the other two repetitions together with a later time of occurrence of the second peak velocity in this repetition. The time of occurrence of the minimal velocity was later with each repetition in the control condition, while in the eccentric loading condition it only increased from repetition 1 with 3 (Figure 3).

**Figure 4:**
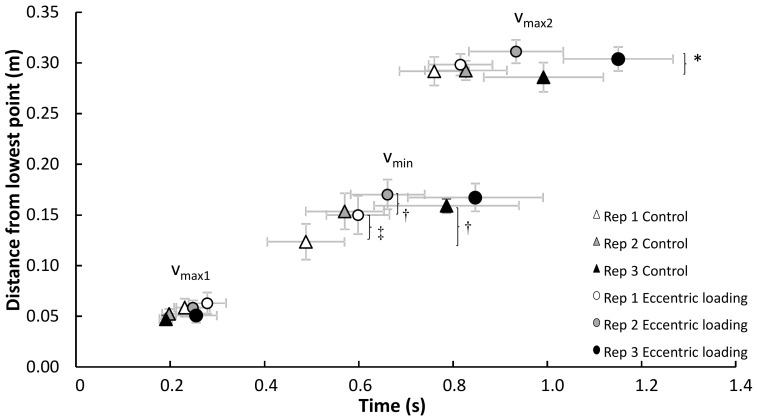
Distance from lowest barbell point (± SEM) at the different events in each repetition for each condition during the concentric phase. * indicates a significant difference in total distance from lowest barbell point to v_max2_ between these two conditions on a *p* < 0.05. † indicates a significant difference in distance travelled from v_max1_ to v_min_ between these two repetitions for this condition at *p* < 0.05. ‡ indicates a significant difference in distance travelled from v_max1_ to v_min_ between both conditions for this repetition at *p* < 0.05.

A significant effect of condition on barbell distance was found for the distance traveled from v_max1_ to v_min_ and the total distance traveled from v_0_ to v_max2_ (F ≥ 5.2, *p* ≤ 0.046, η^2^ ≥ 0.34). Only a significant effect of repetition was found for distance traveled from v_max1_ to v_min_ (F = 8.6, *p* = 0.002, η^2^ = 0.46). Post hoc comparisons revealed that barbell distance travelled more in the augmented eccentric loading from v_max1_ to v_min_ than the control condition, but it was only significant for repetition 1 between the two conditions (*p* = 0.034). In the control condition, the travelled distance increased significantly from repetition 1 with 3, while in the augmented eccentric loading condition it only increased from repetition 1 with 2. The total barbell distance travelled from v_0_ to v_max2_ was longer in the augmented loading compared with the control condition; however, the difference was only significantly longer in repetition 2 between the two conditions (*p* = 0.029; Figure 4).

Only a significant effect of condition was found in the sternal part of the pectoralis major in the eccentric phase and for the biceps in the sticking region. Post hoc comparison showed that biceps activation was higher in the augmented loading condition during the sticking region, while higher activation occurred in the sternal part of pectoralis major during the eccentric phase of the control condition compared with the other condition (Table 1). Furthermore, a significant effect of repetition was found for several muscles in different regions (see Table 1 for details). In addition, a significant interaction effect (condition*repetition) was found for the anterior deltoid and lateral triceps in the sticking region, and for the lateral triceps, posterior deltoid and clavicular part of pectoralis major in the post-sticking region. The anterior deltoid increased activation during the three repetitions in the sticking region in the control condition, while activation was similar across the three repetitions in the augmented eccentric loading condition. In the post-sticking region, the lateral triceps muscle and the posterior deltoid muscle increased more in activation during the three repetitions in the control condition compared with the augmented eccentric condition resulting in a significantly higher activation in repetition 3 for these two muscles. While for the clavicular part of the pectoralis major activation started lower at repetition 1 in the augmented eccentric condition compared with the control condition and increased to a similar activation level as the control condition in repetition 3 (Table 1).

## 4. Discussion

The aim of this study was to investigate the effects of an augmented eccentric loading upon the kinematics and muscle activation that occur during bench press. The main findings of this study were that only kinematic differences between the augmented and control condition were found in the eccentric phase with a lower peak velocity in the augmented eccentric condition. In the concentric phase, only an effect of repetition was found. Furthermore, the augmented loading condition caused a higher activation of the biceps during the pre-sticking and sticking region, while a lower activation in the sternal part of pectoralis major occurred during the eccentric phase compared with the control condition.

The finding of our study was that an augmented load of 95% of 1RM in the eccentric phase did not contribute to enhancing the concentric phase, while lifting 85% of 1RM is in accordance with earlier findings of Wagle et al. [25,26] in squats and Ojasto; Hakkinen [16] in bench press who also did not find any enhanced performance while lifting at 1RM with augmented loads of 105, 110, and 120% of 1 RM during the eccentric phase. This was in contrast with the studies of Doan; Newton; Marsit; Triplett-McBride; Koziris; Fry; Kraemer [14] and Sheppard; Young [15] who demonstrated that concentric force could be enhanced by a greater eccentric load. The possible main reasons for this discrepancy with these studies were the loads used and the testing protocol. Sheppard; Young [15] used very low loads of 40–50% of 1RM in the concentric part and had 10–20 kg higher loads in the eccentric phase. This large difference in load between the eccentric and concentric phase seemed to have a positive effect compared to loads at submaximal levels, such as 85–95% of 1RM or 1RM loads [16]. Doan; Newton; Marsit; Triplett-McBride; Koziris; Fry; Kraemer [14] used 1RM (eccentric phase +5%) loads but used a different testing protocol in which the subject had three attempts where the 1RM load was increased after each successful attempt.

Doan; Newton; Marsit; Triplett-McBride; Koziris; Fry; Kraemer [14] gave four possible explanations for their results: (1) an increase in neural stimulation, (2) recovery of stored elastic energy, (3) contractile machinery alterations, and (4) increased preload, without any measurements that could confirm this. However, in the present study the higher eccentric load resulted in a lower peak velocity. This lower peak velocity was the result of a lower acceleration due to the higher load. Based on this evidence, we speculate that the total lowering force was the same in both conditions as both attempts were performed with maximal effort [27]. Thereby the preload was the same between the two conditions. An augmented eccentric load would also result in a slower eccentric portion of the lift, thus eliminating the effect of a stretch shortening cycle as suggested by Bobbert; Gerritsen; Litjens; van Soest [5]. In fact, previous studies reported that a faster eccentric portion led to greater concentric performance as measured by average and peak concentric velocity, suggesting that eccentric velocity could have a causal role in subsequent concentric performance [28,29].

Furthermore, no differences in the first peak velocity and its occurrence (pre-sticking region) were found between the two conditions, indicating that the augmented eccentric load did not have a direct potentiation effect (the first 0.3 s) upon the concentric part of the bench press (Figure 3). However, augmented eccentric loading seemed to have an effect in the sticking region shown by more barbell distance in this region compared with the control condition. This also resulted in more total distance at v_max2_ (Figure 4). However, these differences in distance cannot be explained by differences in peak and minimal velocities between the two conditions (Figure 3). It is suggested that the duration of the sticking region could be the cause for the difference in total barbell distance. On average, the duration of the pre-sticking and sticking region were 0.1 s longer (0.7 vs 0.6 s) after augmented eccentric loading, which did not reach statistical significance (*p* = 0.066) due to variability within subjects (Figure 3). Thereby, it seems that augmented eccentric loading did not cause more potentiation, but more fatigue [18], as visible by lower velocities and longer duration of events (Figure 3).

It was hypothesised that augmented eccentric loading would cause more pre-activation, especially in the eccentric phase of the lift, which did not occur. In general, no significant differences were found between the two conditions in the eccentric phase except for the sternal part of the pectoralis major, which was even lower with the augmented eccentric loading condition. This indicated that this extra 10% augmented loading is not enough to cause extra pre-activation as was expected. Probably more load needed to be added to cause a difference, as shown in the study of Sheppard; Young [15]. This was also visible in muscle activity during the concentric phase, as the muscle activity of most muscles was similar among the conditions. Only the biceps during the pre-sticking and sticking region showed a significant difference during the first repetition. The biceps acts as a stabiliser during bench press [30] and the higher activity in the augmented eccentric condition could be due to the requirement of stabilisation of the elbow joint during the first repetition. In the first repetition in particular, this extra stability could be necessary because, in the first repetition, the subject always has to explore his own performance, when several repetitions are involved [31]. In the later repetitions the subject is aware of his performance for that day and needs less stability from the biceps [18]. Furthermore, the 10% extra load in the eccentric phase causes a perturbation at the lowest barbell point (losing the hook with the extra load), which means more stability requirements, especially in the first repetition. Thereby, activating the biceps more in the start of the concentric phase. This perturbation could perhaps also explain the absence of an improved subsequent concentric phase.

An interaction effect was found in the post-sticking region for the lateral triceps muscle, the posterior deltoid muscle and clavicular part of the pectoralis major in which muscle activation was higher in the control condition compared with the augmented eccentric loading (Table 1). This indicates that augmented eccentric loading has a certain effect upon the concentric phase during the lift, but not in the start of the concentric phase as expected. It seemed to cause less fatigue to these muscles later in the movement, which could be a positive finding when including this extra eccentric load for training purposes. However, more studies should be conducted, with different augmented eccentric loads before it is possible to point out the acute effect of augmented eccentric loading and the underlying mechanisms behind this type of training. Therefore, future research, should perform augmented eccentric loading over a chronic period of time to investigate if it could potentially lead to greater adaptations in strength and power.

## 5. Conclusions

Based upon the findings of the present study, it can be concluded that augmented eccentric loading with 95% of 1RM in bench press did not have any acute positive effect upon the concentric phase of the lift at 85% of 1RM and that the proposed underlying mechanisms, such as potentiation, increased neural stimulation, preload, and recovery of stored elastic energy, did not seem to occur with these loads.

## Figures and Tables

**Table 1:** Mean RMS muscle activity (± SEM) of the different muscles in the different regions during the augmented eccentric loading (Ecc. L) and control condition (Cond.).

Muscle	Cond.	Eccentric Phase			Pre sticking Region			Sticking Region			Post Sticking Region		
		Rep 1	Rep 2	Rep 3	Rep 1	Rep 2	Rep 3	Rep 1	Rep 2	Rep 3	Rep 1	Rep 2	Rep 3
Anterior deltoid	Control	409 ± 54 *	457 ± 55	455 ± 56	611 ± 70	658 ± 79	739 ± 97 *	634 ± 78	674 ± 66	784 ± 86 *	684 ± 64 *	752 ± 74	812 ± 66
	Ecc. L.	452 ± 51	480 ± 55	507 ± 56†	694 ± 64	725 ± 75	781 ± 87†	717 ± 76	740 ± 80	744 ± 87	682 ± 72 *	739 ± 75	771 ± 74
Lateral deltoid	Control	142 ± 33 *	163 ± 38	164 ± 40	197 ± 41	206 ± 37	242 ± 51†	222 ± 51	235 ± 50	257 ± 52	215 ± 40	236 ± 42	243 ± 41
	Ecc. L.	161 ± 33	167 ± 32	167 ± 33	203 ± 34	214 ± 38	220 ± 37	217 ± 40	249 ± 44	279 ± 49	226 ± 36	243 ± 41	246 ± 41
Posterior deltoid	Control	111 ± 47 *	131 ± 54	133 ± 54	98 ± 13	112 ± 13	122 ± 20	113 ± 14 *	120 ± 16	138 ± 20	97 ± 9 *	123 ± 13	139 ± 17
	Ecc. L.	87 ± 14	92 ± 16	91 ± 15	87 ± 7 *	109 ± 12	115 ± 16	100 ± 9	121 ± 14	120 ± 13	104 ± 11 *	114 ± 12	123 ± 16
Lateral triceps	Control	525 ± 85	542 ± 86	540 ± 78	732 ± 127	846 ± 114	819 ± 121	758 ± 116	787 ± 110	890 ± 120 *	676 ± 102	763 ± 116	854 ± 123 *
	Ecc. L.	567 ± 70 *	618 ± 81	635 ± 78	751 ± 121 *	823 ± 104	891 ± 142	769 ± 126 *	847 ± 113	853 ± 117	758 ± 114 *	783 ± 112	780 ± 113
Long head triceps	Control	16 ± 2	16 ± 3	18 ± 4	31 ± 5	31 ± 6	33 ± 8	39 ± 7	38 ± 6	40 ± 6	36 ± 5	41 ± 5	41 ± 4
	Ecc. L.	15 ± 2	15 ± 2	16 ± 3	25 ± 3	30 ± 5	31 ± 7	27 ± 3 *	37 ± 5	36 ± 3	34 ± 4	38 ± 4	36 ± 4
Sternal part pectoralis major	Control	252 ± 33 *	284 ± 37 *	310 ± 44 *	365 ± 53	392 ± 44	448 ± 66	394 ± 70	430 ± 71	506 ± 84 *	394 ± 59	397 ± 64	437 ± 68 *
	Ecc. L.	221 ± 33 *	256 ± 37 *	272 ± 40 *	334 ± 52	368 ± 59	450 ± 72 *	352 ± 67	412 ± 68	449 ± 75	335 ± 58 *	417 ± 60	421 ± 60
Clavicular part pectoralis major	Control	283 ± 45	304 ± 42	326 ± 53†	513 ± 105	551 ± 123	569 ± 111	440 ± 61	478 ± 70	540 ± 59†	446 ± 50	437 ± 55	515 ± 62 *
	Ecc. L.	297 ± 38 *	328 ± 40	335 ± 39	523 ± 95	550 ± ±121	581 ± 100	446 ± 61	528 ± 78	467 ± 63	388 ± 51 *	456 ± 60 *	537 ± 74 *
Biceps	Control	149 ± 51 *	182 ± 58	203 ± 65	80 ± 19	124 ± 37	127 ± 47	40 ± 9	49 ± 10	55 ± 13†	40 ± 9 *	46 ± 10	52 ± 13
	Ecc. L.	244 ± 76	220 ± 72	249 ± 80	211 ± 50	191 ± 56	208 ± 64	142 ± 43	67 ± 11	76 ± 23	52 ± 7	53 ± 9	57 ± 9

* indicates a significant difference with the other repetitions on a *p* < 0.05 level. † indicates a significant difference with the repetition 1 on a *p* < 0.05 level. Red colour indicates a significant difference with the other condition for this repetition on a *p* < 0.05 level. Blue colour indicates rep*condition interaction effect and significant difference between the two conditions at this repetition on a *p* < 0.05 level.
